# THREAT helps to identify epistaxis patients requiring blood transfusions

**DOI:** 10.1186/1916-0216-42-4

**Published:** 2013-01-31

**Authors:** Karin Murer, Nader Ahmad, Beatrice A Roth, David Holzmann, Michael B Soyka

**Affiliations:** 1 Department of Otorhinolaryngology, Head and Neck Surgery, Kantonsspital St. Gallen, St. Gallen, Switzerland; 2 Department of Otorhinolaryngology, Head and Neck Surgery, University Hospital Zurich, Zurich, 8091, Switzerland; 3 Swiss Federal Veterinary Office, Zollikofen, Switzerland

**Keywords:** Epistaxis, Anemia, Blood transfusion

## Abstract

**Objective:**

To analyze the characteristics of patients who needed a blood transfusion due to epistaxis-caused anemia and to define potential risk factors.

**Design:**

Retrospective cohort study.

**Setting:**

A total cohort of 591 epistaxis patients, prospectively included between March 2007 and April 2008 at the ENT department of the University Hospital of Zurich, was evaluated concerning the need for blood transfusions.

**Methods:**

The clinical charts and medical histories of these patients were evaluated.

**Main outcome measures:**

Common parameters that increase the risk for severe anemia due to epistaxis.

**Results:**

Twenty-two patients required blood transfusions due to their medical condition. 22.7% suffered from traumatic nosebleeds. Another 27.3% had a known medical condition with an increased bleeding tendency. These proportions were significantly higher than in the group of patients without need of blood transfusion. The odds ratio for receiving a blood transfusion was 14.0 in patients with hematologic disorders, 4.3 in traumatic epistaxis and 7.7 in posterior bleeders. The transfusion-dependent epistaxis patients suffered significantly more often from severe posterior nosebleeds with the need for a surgical therapeutic approach.

**Conclusions:**

Patients with severe nosebleeds either from the posterior part of the nose or with known hematologic disorders or traumatic epistaxis should be closely monitored by blood parameter analyses to evaluate the indication for hemotransfusion. The acronym THREAT (Trauma, Hematologic disorder, and REAr origin of bleeding → Transfusion) helps to remember and identify the factors associated with an increased risk of receiving blood transfusion.

## Background

Epistaxis is the most common emergency in rhinology and accounts for almost six hundred consultations at the University Hospital of Zurich per year [1]. Most commonly the bleeding originates from the anterior part of the nose and can easily be controlled by chemical or electrical cautery or anterior packing. On the other hand, epistaxis can also be severe, requiring hospitalization and “aggressive” management including repeated nasal packing, surgery or arterial embolization [2-4]. Severe epistaxis is usually posterior in origin. Some patients even require blood transfusions due to their high level of blood loss. A predominantly benign disorder can therefore also become life-threatening. Blood transfusions must be considered if hemoglobin levels around 7 g/dl are present. If normovolemia cannot be maintained, the patient is critically ill or the patient has other co-morbidities (e.g. cardiovascular and chronic pulmonary disease, receiving chemotherapy etc.) a blood transfusion has to be considered earlier and despite higher hemoglobin levels [5-7].

Epistaxis may be posttraumatic, iatrogenic (after endonasal surgery) or “spontaneous”. Numerous causative factors such as local nasal inflammation, medication, platelet and coagulation abnormalities, alcoholism and hereditary hemorrhagic telangiectasia have been suggested [8]. For a long time, hypertension was considered to be a major cause of spontaneous epistaxis, however this topic appears to be more controversial in recent literature [9-13].

The aim of this retrospective study was to investigate the small population of patients who needed blood transfusions during their epistaxis treatment and to search for common factors that may represent risk factors. For this reason a retrospective analysis of a large study population was conducted. To our knowledge this is the first report of this topic in the literature.

## Methods

### Patients

Patients presenting at the ENT department of the University Hospital of Zurich for the reason of epistaxis were prospectively included in a previous study [1] between March 2007 and April 2008. As the original prospectively recorded data contained information on systemic disorders and the need for blood transfusion, it was possible to further investigate those patients. We therefore retrospectively analyzed the data with particular interest in patients who required blood transfusions. All patients were divided into two groups: those requiring a blood transfusion during their epistaxis treatment and those who did not receive any blood-products. Multiple factors influence the decision of giving blood to a patient during the treatment of epistaxis. In our clinic the administration of blood transfusions is evaluated with respect to the patients’ hemoglobin levels, symptoms (low blood pressure, tachycardia, dizziness) and medical co-morbidities such as a high risk for cardiac infarction. As this study was conducted retrospectively we did not define clear cut-off values or conditions for the need of hemotransfusion. The criteria are further reviewed in the discussion section.

The lowest hemoglobin level before transfusion, site of bleeding, history of hematologic or vascular disorder, history of acute nose trauma, exposure to anticoagulants and blood platelet antiaggregant medications as well as the type of treatment were evaluated for their association with the need for blood transfusions. Patients were asked about their history of hypertension to find a possible link to an increased risk of blood transfusion. All patients who confirmed suffering from arterial hypertension were taken into the “positive hypertension-history” group even if blood pressure was well-adjusted with medication at the moment of presentation.

Patients with epistaxis due to trauma with relevant blood loss from additional sites of the body, other than the nose itself, were excluded. A nose trauma was defined as a direct or indirect injury of nasal/paranasal structures with a consecutive nosebleed, visible bruise marks, scars or nasal bone fracture at presentation. Nose picking was not considered a nasal trauma, as its relevance in epistaxis remains questionable [14].

Posterior bleeding was defined as a bleeding source not visible upon anterior rhinoscopy.

The treatment plan at our department has previously been published [1] and evaluated [15]. In summary, the treatment of choice for anteriorly located bleeding is electric or chemical cautery. For posterior epistaxis a nasal endoscopy is performed in surface anesthesia to localize and if possible cauterize a bleeding source. As this is rarely possible we usually place an inflatable Rapid Rhino® packing (7.5 cm anterior-posterior, ENT Arthrocare Europe, Stockholm, Sweden) in posterior bleeds. If this treatment fails we insert a Foley balloon catheter and pack the nose with fat-gauze. In case of repeated failure or if a new bleed arises after removing of the packing, a rigid nasal endoscopy is performed in general anesthesia and all branches of the sphenopalatine artery are coagulated and transected. If the bleeding persists or the source of bleeding is clearly located in the supply area of the ethmoidal arteries, external closure is performed by lynch incision or transcaruncular approach [16].

### Ethical consideration

Data collection was performed with the permission of the local ethical committee and review board.

### Statistical analysis

Comparisons between the groups were performed with a Chi-Square test for categorical variables. For the multivariate analysis a logistic regression model using a step-wise backward method was calculated using R [17] (version 2.14.1). The explanatory variables bleeding localization (anterior/posterior), trauma (yes/no), exposure to anticoagulants or platelet antiaggregants (yes/no), history of hypertension (yes/no) and history of hematologic or vascular disorder (yes/no) were included in the full model. The 5% significance level was applied as a threshold for exclusion of explanatory variables from the model and for other statistical tests. Apart from the regression analysis, all other statistical computations were performed using Prism Version 5 (GraphPad Software, USA).

## Results

Between March 2007 and April 2008, 591 individuals were seen at the ENT department of the University Hospital of Zurich for the reason of epistaxis. In the literature this is one of the largest epistaxis populations that have been investigated.

Twenty-two patients (3.7%) needed blood transfusion due to their medical condition. Table 1 shows the characteristics of these 22 patients in comparison to the group of epistaxis patients without blood transfusions. Table 2 indicates hemoglobin values and number of blood transfusions in this small patient group. Age and gender distribution was equal in both groups. 72.7% of the blood transfusion group presented with posterior epistaxis. This percentage is significantly higher than in the group without blood transfusion (p < 0.01), therefore the treating modalities where also significantly different. Surgical management of epistaxis with endoscopic closure of the sphenopalatine artery or open ligature of the ethmoidal arteries was needed in 31.8% of patients in the blood transfusion group, whereas in patients without blood transfusions only 5.5% had a surgical intervention (p < 0.01). Only 5 patients (0.85%) received open ligature of the ethmoidal arteries. One of these 5 required blood transfusion.

**Table 1:** Patients characteristics

		**Blood transfusion Yes, n = 22, 3.7%**		**Blood transfusion No, n = 569, 96.3%**		***p***
**Variable**	**Value**	**Number**	**%**	**Number**	**%**	
Sex	male	11	50	320	56.2	0.7
	female	11	50	249	43.8	
Bleeding Localization	anterior	5	22.7	402	70.7	<0.01
	posterior	17	77.3	167	29.3	
Trauma	Yes	5	22.7	30	5.3	<0.01
	No	17	77.3	539	94.7	
Platelet antiaggregant medications	Yes	9	40.9	207	36.4	0.7
	No	13	59.1	362	63.6	
Oral anticoagulants	Yes	2	9.1	114	20	0.3
	No	20	90.9	455	80	
History of hypertension	Yes	12	54.5	317	55.7	1
	No	10	45.5	242	42.5	
Known hematologic and vascular disorders	Yes	6	27.3	19	3.3	<0.01
	No	16	72.7	550	96.7	
Elevated liver enzymes	Yes	5	22.7	98	17.2	0.6
	No	17	77.3	471	82.8	
Therapy (>1 modality per patient possible)	electric or chemical cautery	7	31.8	483	84.9	<0.01
	Nasal packing	14	63.6	197	34.6	0.01
	Operation	7	31.8	31	5.5	<0.01
First bleeding episode at presentation	Yes	5	22.7	176	30.9	0.5
	No	17	77.3	393	69.1	
Hospitalisation	Yes	19	86.4	80	14.1	<0.01
	No	3	13.6	489	85.9	

**Table 2:** Transfusion related characteristics of patients

		**Blood transfusion Yes, n = 22, 3.7%**		**Blood transfusion No, n = 569, 96.3%**	
**Variable**	**Value**	**Range**	**Median**	**Range**	**Median**
Age	years	20-95	72	12-97	69.5
Blood Transfusions	Number of units	1-6	2	-	-
Hemoglobin level before Transfusion	g/l	48-95	73.5	-	-

A multivariate analysis including the variables “localization of the bleeding, trauma, antiaggregational medication, anticoagulants, history of hypertension and hematologic or vascular disorders” revealed an increased risk for the administration of hemotransfusions. In the final model a significantly increased risk for hemotransfusions was found for posterior bleedings (p < 0.01) with an odds ratio (OR) of 7.7 (95%-CI 2.8/24.7), hematologic or vascular disorders (p < 0.01) with an OR of 14.0 (95%-CI 4.1/45.7) and trauma (p = 0.02) with an OR of 4.3 (95%-CI 1.2/45.7). Doing a subgroup analysis on patients who were on antiaggregational medication only the localization of the bleeding remained in the model. A significantly higher proportion of posterior epistaxis (p < 0.01) was found showing an OR of 26.3 (95%-CI 4.9/485.9). Subgroup regression analyses within the group of posterior bleeders revealed an increased risk of blood transfusions when hematologic disorders were present OR 6.0 (p = 0.02) and a trendwise increased risk in trauma patients OR 3.2 (p = 0.07).

Of the 22 patients in the blood transfusion group, 6 patients (27.3%) suffered from hematologic or vascular disorders, namely: hereditary hemorrhagic telangiectasia (Osler-Weber-Rendu disease), recurrent thrombocytopenia of unknown etiology, myelodysplastic syndrome, non-hodgkin lymphoma, splenomegaly with thrombocytopenia due to non-hodgkin lymphoma and multiple myeloma. Three patients (13.6%) suffered from other severe medical preconditions, 6 (27.3%) had recurrent nosebleeds requiring several interventions. Only 2 (9%) had no special risk factors besides taking anticoagulants or antiaggregational medication. Taking together all patients with either traumatic epistaxis or hematologic/systemic medical disorders we found a sensitivity of 64% and a specificity of 84% when testing for transfusion requirement, with a negative predictive value of 98%.

Patients in the blood transfusion group had a similar exposure to anticoagulants and platelet antiaggregant medications as patients without transfusions. A correlation between the intake of these medications and the need of a blood transfusion could not be shown. There was also no difference in the appearance of elevated liver enzymes between the two groups. A known history of hypertension was present in approximately half of the patients in both groups.

The 22 patients with a need for blood transfusions received on average 2.6 units per patient (range 1–6, median 2). The lowest hemoglobin value measured before transfusion ranged between 48 and 95 g/l (median 73.5 g/l).

The hospitalization rate was 16.8% in the study population with 99 patients spending at least one night in hospital. In the blood transfusion group this rate was significantly higher (p < 0.01) with 86.4% of patients hospitalized staying one night or longer. In the subgroup of hospitalized patients regression analysis showed an increased risk just for patients with hematologic disorders with an OR of 6.8 (p = 0.02). Concerning complications, one patient in the transfusion group suffered from myocardial infarction and had to be transferred to the intensive care unit. None of the patients in either group died as a consequence of epistaxis or its treatment.

## Discussion

In most cases epistaxis is a benign condition, but sometimes it can be serious and become a challenging medical problem in otorhinolaryngology. Some patients with severe epistaxis, usually posterior in origin, required more intensive care. Ninety-nine patients (16.8%) out of our collective of 591 needed a hospitalization. The need for blood transfusion occurred in 22 (3.7%) patients. These 22 patients had some common characteristics. As this study was performed retrospectively we need to be aware of the potential report-bias, where doctors keep better records of “difficult” patients (such as those addressed by this study) than in unproblematic cases. We tried to reduce this bias by including a large number of patients and using the prospective protocol in which data was collected.

Most (77.3%) of the patients in the transfusion group suffered from posterior epistaxis. Posterior localization of bleeding is a predictor of surgical treatment a strong association has been shown between surgery for epistaxis and the need for blood transfusions [18]. Another study reported similar rates of blood transfusion (45.5%) in patients requiring sphenopalatine artery ligation [19]. However this investigation only included 11 patients and the criteria for blood transfusion were not described.

### Transfusion strategy

In our collective, of the subset of patients that required surgical treatment, only 18.4% received blood transfusions. In accordance with current literature, we follow a restrictive transfusion strategy at our institution. The mean transfusion trigger in our patients was a hemoglobin level of 7.4 g/dl. For many years physicians believed that a hemoglobin of 10 g/dl and a hematocrit of 30% represented the goal in anemic patients, especially those undergoing surgery and those with cardiac disease [20]. In the last decade many studies have demonstrated that a substantially lower hemoglobin level (7 g/dl) can be tolerated in patients who are not critically ill, if normovolemia is maintained [5-7]. We also considered blood transfusion if there was an acute severe nosebleed with signs of hypovolemia, identified by tachycardia and low blood pressure or in high-risk patients (e.g. cardiovascular and chronic pulmonary disease, patients receiving chemotherapy etc.) with hemoglobin levels below 9 g/dl. We follow this restrictive transfusion regime because besides the benefits of a blood transfusion, it can also be life threatening and is believed to exert serious adverse effects (e.g. lengthened hospital stay and impaired recovery) [21,22].

### Transfusion group characteristics

The risk of receiving blood products increases sevenfold in patients with posterior bleedings compared to anterior epistaxis. In those receiving antiaggregational medication this risk is further increased to an odds ratio of 26. As shown by multivariate subgroup analysis, only posterior localization of the bleeding source increased the necessity to administer transfusions to patients on acetylsalicylic medication or taking Clopidogrel significantly.

Non-iatrogenic posttraumatic epistaxis was significantly more frequent in the transfusion group with a larger than 4-times increased risk for hemotransfusion. Traumatic injury of a vessel is suspected to be worse than in cases of spontaneous epistaxis. It can be assumed that in traumatic cases spontaneous closure is more difficult and therefore prolonged bleeding with increased blood loss can be expected. As mentioned before we excluded patients with relevant blood loss from additional trauma sites of the body.

Six patients in the transfusion group suffered from hematologic or vascular disorders that are known to have an influence on hemostasis or vessel integrity. These patients have a 14 times higher risk for hemotransfusion.

Hereditary hemorrhagic telangiectasia (Osler-Weber-Rendu disease) is an autosomal dominant disorder characterized by abnormal angiogenesis. The most common manifestation is epistaxis resulting from trauma to thin-walled telangiectasias [23]. Myelodysplastic syndrome is a disorder of hematopoietic stem cells characterized by ineffective hematopoiesis. The result is pancytopenia leading to anemia and an increased risk of infection or bleeding [24]. In multiple myeloma abnormal hemostasis, specifically the imbalance in function of all major components of the coagulation cascade is known [25]. In non-Hodgkin's lymphomas autoimmune thrombocytopenia is a common immune-hematologic complication [26].

Another 3 patients in the transfusion group who had no trauma and no known vascular or hematologic disease were found to have other major medical problems. One patient suffered from alcohol-induced myocardial pathology, another from primary biliary cirrhosis and was immunosuppressed after kidney transplantation and the third had an HIV infection with microcytic hypochromic anemia. Six other patients who needed blood transfusion had multiple treatments with treatment failure in the weeks before presenting at our institution. Only 2 patients who received a blood transfusion had no such risk factors besides Aspirin and Clopidogrel intake.

As the cohort of our epistaxis patients consists of a very heterogeneous collection of patients one could of course argue that risk factors could be better determined in subgroup analyses. However it was not the aim of the study to subdivide the population but much rather to give treating doctors hints on when to be cautious and to be alert of potential complications due to high blood loss. Nevertheless we performed multiple binary logistic regression analyses in the subgroups of posterior bleeders and also in the hospitalized subgroup. In posterior epistaxis patients the risk factors remained the same, being trauma and hematologic disorders. In the hospitalized patients only hematologic disorders stayed in the model and no other risk factors could be identified, which is not surprising. Hospitalization is reversely linked with transfusions as most of the patients stayed in hospital after the administration of blood products, therefore testing in this highly biased subgroup does not yield relevant new information.

### Synopsis of key findings

Based on our results we suggest monitoring all patients with either traumatic epistaxis or known medical and hematologic disorders more closely than others. Taking these parameters into account 64% of all at risk patients will be identified. Including recurrences will increase this number to 91% at the expense of specificity. We are fully aware that the above mentioned risk factors alone will not identify all patients with the need of a transfusion, they however will help clinicians in the decision of whether or not to perform blood testing in questionable situations. There is no substitute for a thorough risk analysis of every individual patient.

## Conclusions

We identified 3 major risk indicators for the need of blood transfusions in our epistaxis cohort, being: Traumatic bleeding, posterior origin of bleeding source and hematologic/vascular disorders. The acronym THREAT (Trauma, Hematologic disorder, and REAr origin of bleeding → Transfusion) helps to remember and identify those risk factors. Patients with severe nosebleeds, especially of posterior origin, who had several bleeding episodes over the time before presenting at our institution, were at increased risk to undergo surgery and also to receive blood transfusions. Our investigation also showed that patients who are known to have hematologic, vascular or other systemic disorders and patients with nosebleeds after trauma should be closely monitored including blood parameter analyses.

## Competing interests

The authors declare that they have no competing interests.

## Authors’ contributions

KM performed the data collection and wrote the manuscript, NA collected the data and critically reviewed the manuscript, BAR performed statistical analyses, DH critically reviewed the study plan and helped in writing the text, MBS has planned the study, helped with data collection and reviewed the manuscript. All authors read and approved the final manuscript.
